# Multi Expression Programming Model for Strength Prediction of Fly-Ash-Treated Alkali-Contaminated Soils

**DOI:** 10.3390/ma15114025

**Published:** 2022-06-06

**Authors:** Kaffayatullah Khan, Mohammed Ashfaq, Mudassir Iqbal, Mohsin Ali Khan, Muhammad Nasir Amin, Faisal I. Shalabi, Muhammad Iftikhar Faraz, Fazal E. Jalal

**Affiliations:** 1 Department of Civil and Environmental Engineering, College of Engineering, King Faisal University (KFU), Al-Ahsa 31982, Saudi Arabia; mgadir@kfu.edu.sa (M.N.A.); fshalabi@kfu.edu.sa (F.I.S.); 2 Department of Civil Engineering, National Institute of Technology Warangal, Warangal 506004, India; gmohdashfaq@gmail.com; 3 Osaimi Geotechnic Company, Tabuk 31952, Saudi Arabia; 4 State Key Laboratory of Ocean Engineering, Shanghai Key Laboratory for Digital Maintenance of Buildings and Infrastructure, School of Naval Architecture, Ocean & Civil Engineering, Shanghai Jiao Tong University, Shanghai 200240, China; mudassiriqbal29@sjtu.edu.cn (M.I.); jalal2412@sjtu.edu.cn (F.E.J.); 5 Department of Civil Engineering, University of Engineering & Technology (UET), Peshawar 39161, Pakistan; 6 Department of Structural Engineering, Military College of Engineering (MCE), National University of Science and Technology (NUST), Islamabad 44000, Pakistan; moak.pg18mce@nust.edu.pk; 7 Department of Civil Engineering, CECOS University of IT and Emerging Sciences, Peshawar 25000, Pakistan; 8 Department of Mechanical Engineering, College of Engineering, King Faisal University (KFU), Al-Ahsa 31982, Saudi Arabia; mfaraz@kfu.edu.sa

**Keywords:** black cotton soil, kaolin soil, alkali contamination, MEP modeling, unconfined compression strength, curing

## Abstract

Rapid industrialization is leading to the pollution of underground natural soil by alkali concentration which may cause problems for the existing expansive soil in the form of producing expanding lattices. This research investigates the effect of stabilizing alkali-contaminated soil by using fly ash. The influence of alkali concentration (2 N and 4 N) and curing period (up to 28 days) on the unconfined compressive strength (UCS) of fly ash (FA)-treated (10%, 15%, and 20%) alkali-contaminated kaolin and black cotton (BC) soils was investigated. The effect of incorporating different dosages of FA (10%, 15%, and 20%) on the UCS_kaolin_ and UCS_BC_ soils was also studied. Sufficient laboratory test data comprising 384 data points were collected, and multi expression programming (MEP) was used to create tree-based models for yielding simple prediction equations to compute the UCS_kaolin_ and UCS_BC_ soils. The experimental results reflected that alkali contamination resulted in reduced UCS (36% and 46%, respectively) for the kaolin and BC soil, whereas the addition of FA resulted in a linear rise in the UCS. The optimal dosage was found to be 20%, and the increase in UCS may be attributed to the alkali-induced pozzolanic reaction and subsequent gain of the UCS due to the formation of calcium-based hydration compounds (with FA addition). Furthermore, the developed models showed reliable performance in the training and validation stages in terms of regression slopes, R, MAE, RMSE, and RSE indices. Models were also validated using parametric and sensitivity analysis which yielded comparable variation while the contribution of each input was consistent with the available literature.

## 1. Introduction

The natural soil–water system can be altered by the interaction of pollutants released from mining, agricultural, and industrial activities. Rapid industrialization has manifested in the form of an exponential rise in the usage of chemical agents which include hydroxides and carbonates and bicarbonates [1,2]. Among the various pollutants, the adverse effects of alkali contamination on the soil–water system have been well established by earlier researchers [3,4]. The lower concentrations of alkali can alter the structure of the soil [5], and at higher concentrations, alkali can augment the formation of new compounds in the form of zeolites and ettringite [4,6]. The alkali interactions with the expansive clays and subsequent swelling are a natural phenomenon primarily driven by the presence of smectite group clay minerals (montmorillonite, illite, and vermiculite) with expanding lattices [5,7]. However, Rao et al. [1] identified the heaving of foundations resting on non-swelling soils contributed by acid and alkali contamination. The incidence of geotechnical failures induced by alkali interaction reported by Rao et al. [1] confirmed the adverse effects of alkali contamination. Traditionally, the swelling of soil was addressed by the utilization of conventional binders such as lime and cement. However, the efficiency of traditional binders was contradicted by Hunter (1990), who reported 3.2 m heave of lime-treated soils. Further, with the growing impetus to promote sustainable binders with relatively lower carbon emissions, the usage of industrial by-products has gained momentum. Among the various industrial by-products, the efficiency of fly ash (FA) as a sustainable stabilizer/binder has been broached by recent researchers [8,9,10,11,12]. Fly ashes inherently do not exhibit unconfined compressive strength (UCS) due to lack/loss of cohesion in either dry or fully saturated conditions [13,14]. However, the UCS of FA is derived either from the difference in capillary action between the coarse and fine fractions [15,16,17] or due to internal friction [18]. Though the studies related to FA treatment on alkali-induced swelling are well documented, the attempts to quantify the variation in strength characteristics are sparse [4,19]. Hence an attempt has been made to evaluate the influence of alkali contamination on the UCS of two types of clay soils, i.e., expansive black cotton (BC) soil and non-expansive kaolin soil, for varying curing periods (1, 7, 14, and 28 days). Further, the effectiveness of FA (10%, 15%, and 20% dry weight) as a stabilizer to enhance the UCS_kaolin_ and UCS_BC_ soils was also evaluated for the aforementioned varying curing periods.

To overcome the limitation of the laborious and time-consuming nature of laboratory studies for the evaluation of engineering characteristics, there is a growing trend of developing numerical models for solving engineering problems [20,21,22] and prediction models for swift estimation of engineering characteristics of soils [23,24]. Initially, most of the prediction models were developed on the basis of regression analysis with relatively limited databases. To overcome this limitation, the advent of artificial intelligence (AI)-based models is being promoted primarily due to their ability to estimate/predict results even for larger databases. Sinha et al. [25] are among the early researchers to introduce artificial neural networks (ANNs), a machine learning language of MATLAB, for the estimation of compaction characteristics for a database of 55 soil samples. The advent of the ANNs has resulted in their consistent usage in dealing with complex physical and mathematical problems [26,27]. Along similar lines, there has been a recent advancement in genetic programming (GP)-based models in the form of gene algorithms (GAs) with the objective of identifying optimized solutions. Cramer (1985) was the first to introduce GP, which was subsequently improved by Koza [28] with varying sizes and shapes. The most advanced methods among the existing linear-based GP techniques are genetic expression programming (GEP) and multi expression programming (MEP); both are genotype computation programming methods that generate tree-like models/programs. The limitations of GAs and GP are addressed by both the methods, which manifests in their efficiency and swift execution (2 to 4 times faster) [29,30,31]. Their unique feature is their ability to learn and adapt by varying their shape, size, and composition, which is akin to living organisms [32,33]. However, unlike GEP, MEP adopts a demonstrative approach and utilizes linear chromosomes for the encoding of programs/solutions. However, the final solution (chromosome) is chosen based on the fitness value of an individual chromosome. In general, the governing parameters in MEP include subpopulation size and number, code length, function set, and crossover probability. Recently, this approach has gained greater applications in the geotechnical engineering field for addressing varied problems which include the prediction of compaction characteristics [34,35], compressive strengths [36,37], permeability and compressibility characteristics [38], deformation modulus [39], soil water characteristic curves, and peak ground acceleration [40]. However, attempts to generate models for the prediction of geotechnical characteristics of the contaminated soils are scarce. Moreover, considering the greater uncertainty associated with clayey soils, there is a need to develop a more realistic MEP model to estimate their strength characteristics. Thus, in the present study, attempts were made to develop an MEP model for the prediction of the UCS of alkali-contaminated clayey soils. For the development of the model, a comprehensive database of 384 soil samples was considered (part of the data was sourced from Ashfaq et al. [19]), and a brief description of the results and the contributing mechanism is presented in the following sections.

## 2. Materials and Methods

### 2.1. Laboratory Studies

The physical properties of the kaolin and BC soils are presented in Table 1, and it can be noted that inherently both the soils are classified as CH (highly plastic clays). The kaolin soil was commercially procured from Heilen Biopharm Pvt. Ltd. The FA considered in the study is sourced from Kakatiya Thermal Power Station (18°23′00.8″ N; 79°49′33.6″ E) Bhupalpally, Telangana, India, and its mineral composition is presented in Table 2 and is classified as class F (due to <30% CaO content). On the other hand, the BC soil was procured from the National Institute of Technology, Warangal (campus), by open excavation at a depth of 1 m (soil profile studies confirmed the presence of BC soil up to a depth of 1.8 m from the ground level), as shown in Table 1. The samples were oven-dried, and a 0.425 mm passing fraction was considered for the UCS tests. Firstly, the soil samples were inundated at varying concentrations of alkali for predetermined curing periods followed by UCS testing. Prior to the UCS testing, the samples were compacted to 95% of MDD of the respective soils, and the strain rate of 0.35 mm/min was applied in accordance with ASTM D2166. To maintain consistency and repeatability, the alkali dosage was maintained at OMC (presented in Table 3) for all the soil–alkali combinations. For the stabilized case, varying dosages of FA (10%, 15%, and 20% by dry weight) were added to the alkali–soil combinations.

### 2.2. MEP Model Development

As stated earlier, the MEP approach is among the most significant linear configurations of the genetic programming (GP) series since it has the ability to deliver simplistic mathematical formulae to forecast a particular prediction model [35,41]. Therefore, the formulization of the UCS_kaolin_ and UCS_BC_ soil was performed in the Multi-Expression Programming X (MEPX version 2021.08.28.0-beta) by incorporating experimental records, as shown in Table 3. Sufficient laboratory test data of 384 different soils for the UCS prediction of FA-treated alkali-contaminated soils were collected by performing an experimental study [42]. The MEP genes are the substrings of varying lengths that keep the chromosomal length constant and equivalent to the total genes on each chromosome. Each gene provides instructions for making a function or a terminal sign, whereas a gene encoding a function includes addresses to the function parameters. The function arguments always have lower parameter estimates than the location of the function on that chromosome [41]. A detailed methodology for generating equations is provided here, and details of the advanced GP approach (MEP simulation) can be found elsewhere [34,35,39,42,43,44,45].

**Figure 1:**
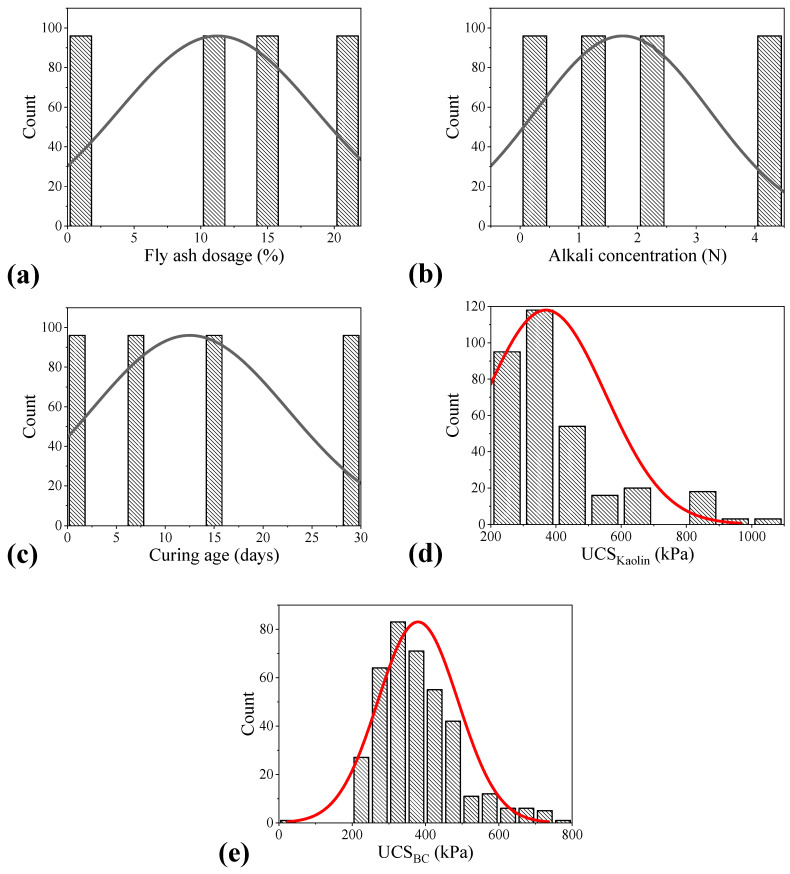
Frequency histograms of the input and output parameters: (**a**) fly ash dosage (%), (**b**) alkali concentration (N), (**c**) curing age (days), (**d**) UCS_kaolin_, and (**e**) UCS_BC_.

Two-thirds of the entire data was considered for the MEP model development, whereas one-third was utilized to validate the formulated model. Table 4 shows the maximum and minimum values of the input (FA dosage, alkali concentration, and curing days) and output parameters (UCS_kaolin_ and UCS_BC_) used to perform strength prediction of FA-treated alkali-contaminated soils in the case of both training and testing data. The maximum as well as the minimum values of all the input and output characteristics have been tabulated in Table 4. Figure 1 shows the frequency histograms (i.e., the scatter of the data) of the input attributes considered in the current study. The curves are smooth and uniformly distributed, which shows a good type of data. In addition, standard deviation (SD), kurtosis, and skewness for all the parameters are given. A smaller SD shows that the parameters are near the respective average value. The kurtosis value represents the sharpness of the peak of a frequency distribution curve. It clarifies the shape of probability distribution [34]. It is pertinent to mention that the kurtosis value is only useful when used in conjunction with the SD value. It is possible that an attribute might have a high kurtosis (bad), but the overall standard deviation is low (good). A kurtosis value of ±1 is considered very good for most psychometric uses, but ±2 is also usually acceptable. The kurtosis values of FA dosage, alkali concentration, curing days, and UCS_kaolin_ approach zero and therefore represent a mesokurtic distribution which can be seen in the histogram plot, i.e., Figure 1. However, the kurtosis value in the case of UCS_kaolin_ is comparatively higher, which represents a leptokurtic distribution (Figure 1). Lastly, the skewness depicts the extent to which a distribution of values deviates from symmetry around the mean. Bryne (2010) argued that data are considered to be normal if skewness is between −2 and +2.

Furthermore, Table 5 shows the Pearson correlation coefficient values (represented by ‘r’) for the input parameters and the two output parameters, i.e., UCS_kaolin_ and UCS_BC_. It can be seen that the impact of all the three input parameters is linearly increasing (because r-values are positive). In the case of both UCS_kaolin_ and UCS_BC_, the order of increasing impact of parameters follows the order: FA dosage > alkali concentration > curing age.

**Figure 2:**
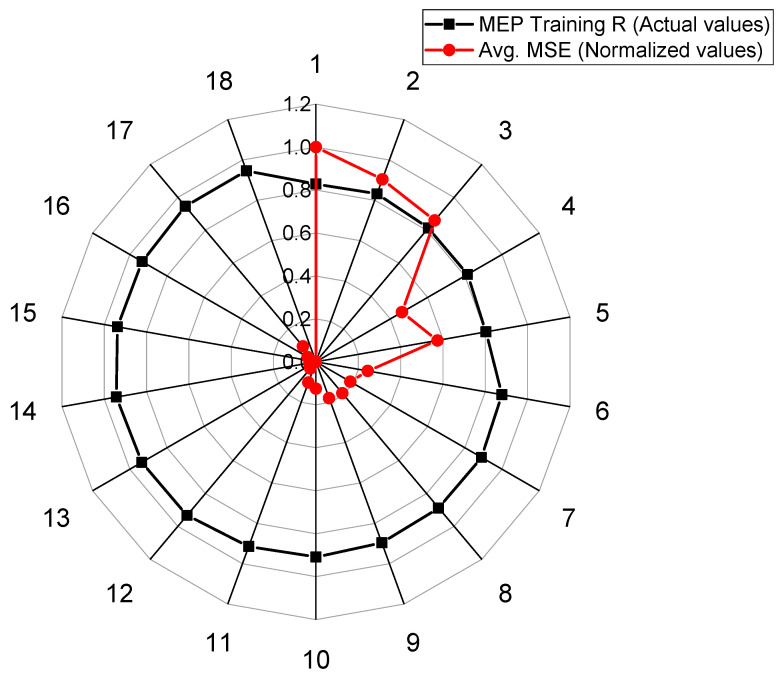
Comparison of normalized averaged MSE and correlation for the developed MEP model for kaolin soil.

**Figure 3:**
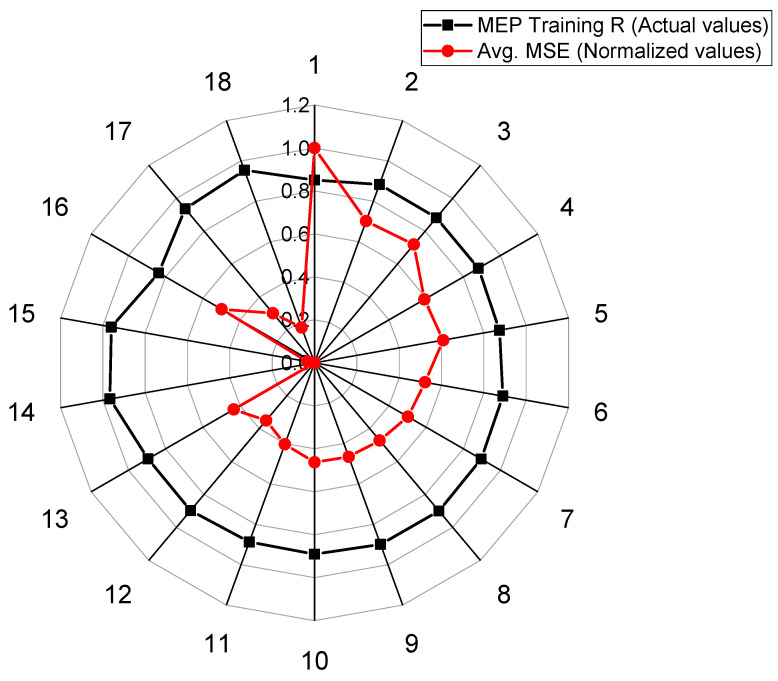
Comparison of normalized averaged MSE and correlation for the developed MEP model for BC soil.

The details of 36 different trials (18 for each type of soil) undertaken to develop a model for the UCS_kaolin_ and UCS_BC_ with an optimal combination of hyperparameters are provided in Table 6. The set of hyperparameters (i.e., number of subpopulations, size of subpopulations, code length, tournament size, and number of generations) was varied in this study to achieve the optimal performance of models. A single parameter was modified whereas the rest were kept unchanged (as shown in Table 7) in an attempt to investigate the effect of different code settings on the correlation coefficient (R) and the mean squared error (MSE), as shown in Figure 2 and Figure 3, respectively.

In order to evaluate the UCS_kaolin_, the best performance was noted in the case of Trial 18 (R = 0.9465, Averaged MSE = 1245), wherein the number of subpopulations, size of subpopulation, code length, number of generations, and tournament size were kept as 20, 1000, 100, 150, and 6, respectively. On the other hand, in determining the UCS_BC_, the best performance was noted in the case of Trial 14 (R = 0.9672, Averaged MSE = 2220), wherein the number of subpopulations, size of subpopulation, code length, number of generations, and tournament size equal 100, 2000, 80, 150, and 2, respectively.

Using the above-mentioned adjusted hyperparameter setting, the simplified mathematical expressions given for the UCS_kaolin_ (Equation (1)) and UCS_BC_ (Equation (2)) were obtained, via C++ code, in order to predict the targeted UCS.

(1) $${\mathrm{UCS}}_{\mathrm{Kaolin}}=7{\;*\;X}_{0}-10{\;*\;X}_{1}+X_{2}-\frac{\left(5\;*\;\left(X_{0}-X_{1}\right)\right)}{X_{1}+2{\;*\;X}_{2}}+\frac{\left(X_{1}{\;*\;X}_{2}+\frac{\left(2{\;*\;X}_{0}{\;*\;X}_{1}\right)}{X_{2}}\right)}{X_{0}-X_{2}}+{\;X}_{0}{\;*\;X}_{1}+\frac{\left(X_{0}{\;*\;X}_{1}{\;*\;X}_{2}\right)}{8}+258$$

(2) $$\begin{array}{cc} {\mathrm{UCS}}_{\mathrm{BC}}= & 9{\;*\;X}_{0}\;-\;X_{1}\;+\;2{\;*\;X}_{1}*\left(X_{1}+X_{2}+1\right)\;-\;\frac{\left(4\;*\;\left(X_{2}\;+\;1\right)\right)}{X_{0}\;-\;1}+X_{1}*\left(X_{1}+\frac{\left(4\;*\;\left(X_{2}\;+\;1\right)\right)}{X_{0}\;-\;1}+X_{1}*\left(X_{0}\;-\;1\right)\right)\\ & -\frac{\left(X_{0}{\;*\;X}_{1}\right)}{X_{2}}\;+\;162 \end{array}$$

where $X_{0}$, $X_{2}$, and $X_{3}$ represent fly ash dosage (%), alkali concentration (N), and curing period (days), respectively.

## 3. Results and Discussion

### 3.1. Strength Characteristics

#### 3.1.1. Effect of Alkali Contamination

**Figure 4:**
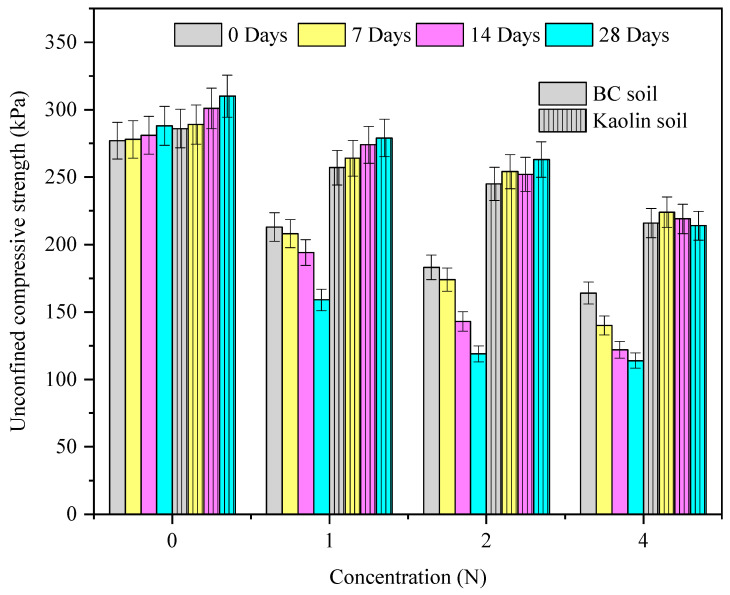
The variation in UCS_kaolin_ and UCS_BC_ with curing period and concentration of alkali after a 28-day curing period.

The variation in the UCS_kaolin_ and UCS_BC_ with curing periods and alkali concentrations is presented in Figure 4. The UCS_kaolin_ and UCS_BC_ linearly decreased with the rise in alkali concentration, and BC soil exhibited a relatively greater fall in the UCS compared to kaolin soil. With the increase in curing periods, both the soils increased linearly for the controlled case. However, under the contaminated case, the kaolin soil exhibited a slight increase in the UCS, whereas the UCS_BC_ remained constant at lower curing periods and was drastically reduced at higher curing periods. The variation in UCS_kaolin_ and UCS_BC_ in the untreated case can be attributed to their inherent mineralogical difference which enables the formation of primary hydration compounds [4]. Under a contaminated scenario, the linear decrease in UCS_kaolin_ and UCS_BC_ soils may be attributed to the increase in charge of clay particles with the pH of the soil. The rise in pH contributes to the subsequent dissolution of silica which varies with the size and crystallinity of quartz, a commonly found mineral in both the soils [4]. Furthermore, an increase in the UCS_kaolin_ with the curing period may be attributed to the precipitation of hydration compounds such as nontronite and sodium silicate hydrate. Similar observations for clayey soils were made by Sivapullaiah and Reddy [4].

#### 3.1.2. Effect of FA Dosage and Curing Period

**Figure 5:**
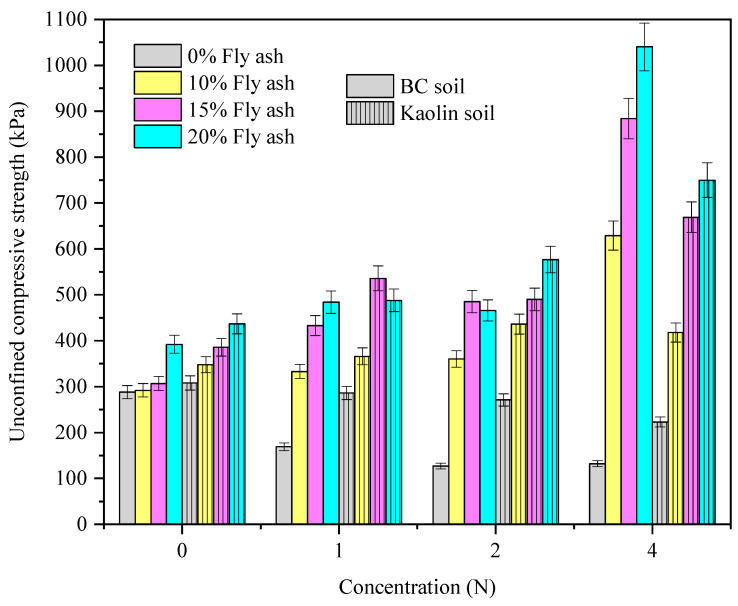
The UCS variation of both the soils with the concentration of alkali and fly ash dosage.

The variation in the UCS_kaolin_ and UCS_BC_ with the alkali concentrations and FA dosage is presented in Figure 5. Considering brevity, the results pertaining to a 28-day curing period have been presented here. It is evident from the results that the FA addition has contributed sufficiently to the linear increase in the UCS_kaolin_ and UCS_BC_ for both the controlled and alkali-contaminated cases. In contrast to the contaminated case, the increase in the UCS_BC_ is substantially higher with an increment of more than 900% compared to the 350% increase noted for kaolin soil. The increase in UCS_kaolin_ and UCS_BC_ is more pronounced at higher concentrations. The linear increase in UCS of both soils is attributed to the decrease in clay content with the FA addition [2]. The greater increment at higher concentration is attributed to the greater affinity of dissolved silica (due to higher concentration of alkali) to react with calcium from FA and subsequent formation of pozzolanic compounds. The pozzolanic compounds formed not only resist alkali attack on mineral phases of soil but also offer greater resistance to compressive loading which is manifested in the form of increased UCS [4,19].

### 3.2. Comparison between Experimental and Predicted Results

This segment focuses on efficacy examination and relative study of the MEP models generated to compute the UCS_kaolin_ and UCS_BC_, using a variety of performance indices. To evaluate the prediction efficiency and accuracy of the proposed MEP models using MEPX software, eight analytical standard indicators, namely regression line slope, correlation coefficient (R), root mean squared error (RMSE), mean absolute error (MAE), root squared error (RSE), relative root mean square error (RRMSE), Nash–Sutcliffe efficiency (NSE) and performance index (ρ) were used in this study [46,47]. These performance measures are defined by the following Equation (3) to Equation (9):

(3) $$\;\;\;\;\;\;\;\;\;\;\;\;\;\;\;\;\;\;\;\;\;\;R=\frac{\sum_{i=1}^{n}\left(E_{i}-{\bar{E}}_{i}\right)\left(P_{i}-{\bar{P}}_{i}\right)}{\sqrt{\sum_{i=1}^{n}{\left(E_{i}-E_{i}\right)}^{2}\sum_{i=1}^{n}{\left(P_{i}-{\bar{P}}_{i}\right)}^{2}}}$$

(4) $$RMSE=\sqrt{\frac{\sum_{i=1}^{n}{\left(E_{i}-P_{i}\right)}^{2}}{n}}$$

(5) $$MAE=\frac{\sum_{i=1}^{n}\left|E_{i}-P_{i}\right|}{n}$$

(6) $$RSE=\frac{\sum_{i=1}^{n}{\left(P_{i}-E_{i}\right)}^{2}}{\sum_{i=1}^{n}{\left(\bar{E}-E_{i}\right)}^{2}}$$

(7) $$RRMSE=\frac{1}{\left|\bar{E}\right|}\sqrt{\frac{\sum_{i=1}^{n}{\left(E_{i}-P_{i}\right)}^{2}}{n}}$$

(8) $$NSE=1-\frac{\sum_{i=1}^{n}{\left(E_{i}-P_{i}\right)}^{2}}{\sum_{i=1}^{n}{\left(P_{i}-{\bar{P}}_{i}\right)}^{2}}\;$$

(9) $$\rho =\frac{\left(\frac{1}{\left|\bar{E}\right|}\sqrt{\frac{\sum_{i=1}^{n}{\left(E_{i}-P_{i}\right)}^{2}}{n}}\right)}{1+R}$$

where E_i_ and P_i_ are the *i*th actual and predicted output values, respectively; ${\bar{E}}_{i}$ and ${\bar{P}}_{i}$ are the average values of the actual and predicted output values, respectively; and n is the number of samples. In addition, the objective function (OBF), as given in Equation (10), shall have a minimum value for better formulation of the model. A smaller OBF helps in overcoming the overfitting problem. A value approaching zero exhibits an excellent predictive capability.

(10) $$OBF=\left(\frac{n_{train}-n_{valid}}{n}\right)\rho_{train}+2\left(\frac{n_{valid}}{n}\right)\rho_{valid}$$

**Figure 6:**
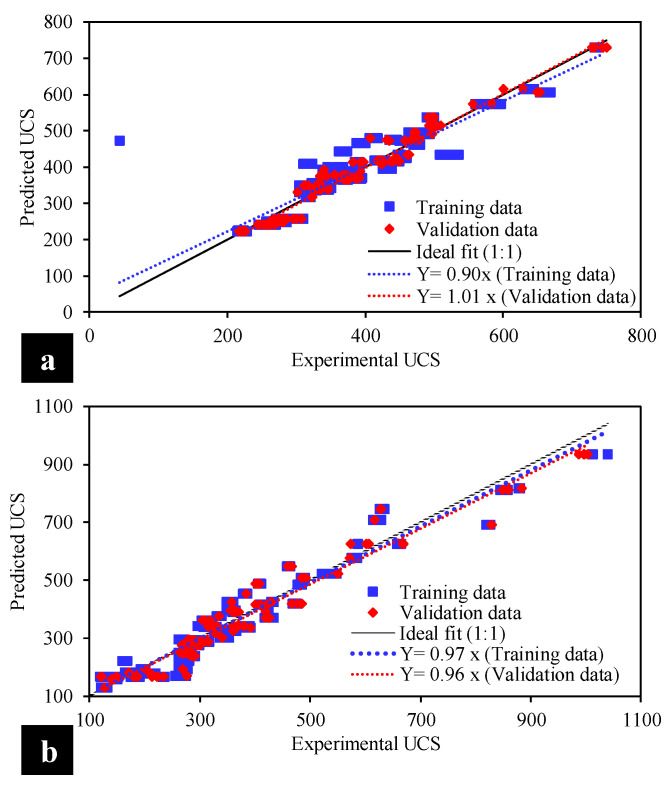
Comparison of experimental and predicted results to evaluate the UCS in the case of (**a**) kaolin soil and (**b**) BC soil.

To construct accurate and robust AI-based predictive models, the ratio of experimental values and inputs in the experimental database (as shown in Table 3) must be greater than 3 and ideally greater than 5 [48]. In the current investigation, the prescribed ratio is 269/3 = 89.66 (training set) and 115/3 = 38.33 (validation set), which is significantly within safe limits and therefore depicts the robustness and superiority of the developed MEP models for the kaolin and BC treated soils. The observed (actual) and forecasted UCS_kaolin_ and UCS_BC_ in the training and validation phases, as well as the efficacy metrics (i.e., slope, R, RMSE, MAE, RSE, RRMSE, and ρ), are shown in Figure 6a,b, respectively. The 45° regression line with a horizontal axis depicts the ideally fit (1:1) line having an inclination corresponding to 1 [49,50]. For good, reliable, and highly correlated models, the dispersion pattern of the data points should be closer to the diagonal line crossing the origin, with a trend line of slope approximately equaling unity, R-value greater than 0.8, and reduced error measurements (i.e., R, RMSE, MAE, RSE, RRMSE, and ρ), as shown in Figure 6 and Table 8. For both the kaolin and BC soil, the slopes of the trend lines are closer to 1 (0.90: training, 1.01: validation; 0.97: training, 0.96: validation, respectively). In addition, the R is above 0.8 (closer to 1) for both types of soils, which reflects a reasonably strong correlation between the model predicted outputs (i.e., UCS_kaolin_ and UCS_BC_) and experimental observations. Furthermore, the OBF value of kaolin soil was 0.025694481, whereas that of BC soil was 0.025050897 in the current study.

Only a higher R-value is not the sole indication of the reliability and accuracy of the machine learning models [34]. Therefore, a number of error measurements were considered to validate the robustness of the developed models. These error metrics include R, RMSE, MAE, RSE, RRMSE, and ρ. The optimizer of the MEP algorithm was set to minimize the MSE while increasing the R statistic. In each model (kaolin or BC soil), the MSE and MAE were relatively low as compared to the maximum expected output, while the RSE reached zero. The optimized UCS_kaolin_ model has MSE and MAE equaling 1245 and 19.6 and 2220 MPa and 30 for the training and validation phases, respectively. Likewise, the discussed attributes are also lower for the optimized UCS_BC_ model. Furthermore, for both optimized models, the RSE tends to approach zero in each phase (i.e., training and validation), confirming their superior functionality. The consistent and accurate performance of the developed models is due to the structural flow of the MEP algorithm. The MEP follows the reproduction procedure to move the relevant information to the subsequent generation and uses the mutation function for optimization inside the chosen chromosomes.

Thus, the predefined configuration of the function is not taken into consideration [51,52]. In addition, the MEP technique produces randomized functions and selects the one that best fits the experimental results [33,40,53].

**Figure 7:**
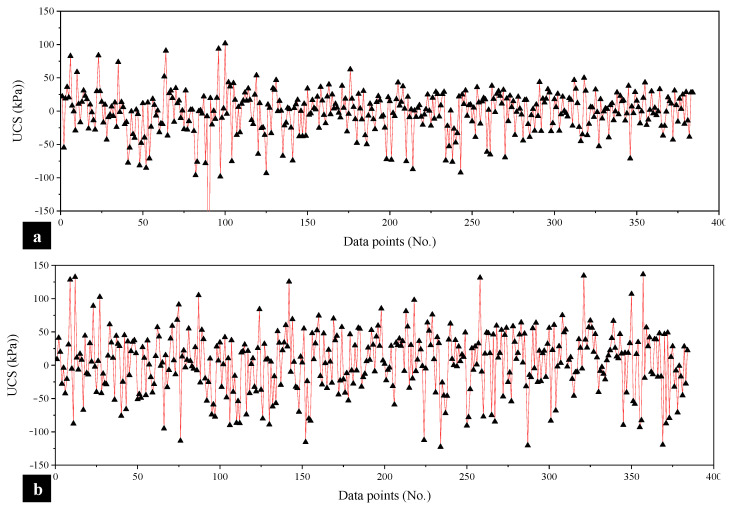
Error analysis of the developed models to evaluate the UCS in the case of (**a**) kaolin soil and (**b**) BC soil.

It is essential to further validate the accuracy of the developed MEP models using the values of the residual error, i.e., the difference between the model-estimated and experimental UCS [54,55]. The positive/negative minimum and maximum error obtained for the UCS_kaolin_ model (Figure 7a) are −160 kPa and 100 kPa, respectively, and are ±130 kPa for the UCS_BC_ model (Figure 7b). The majority of the error readings run along the *x*-axis, indicating a significant frequency of low error values. In conjunction with significantly higher correlations and reduced error measurements, the proposed models could be advantageously employed for the prediction of UCS_kaolin_ and UCS_BC_, assisting practitioners and designers to save time and skip costly laboratory tests.

**Figure 8:**
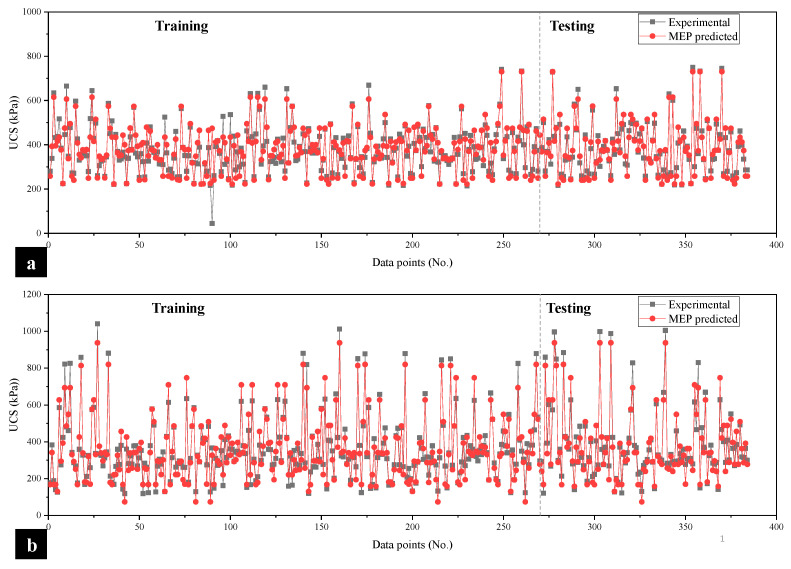
Tracing of experimental results by predicted values to evaluate the UCS in the case of (**a**) kaolin soil and (**b**) BC soil.

The plot of actual experimental values and the ultimate response of the MEP model for estimation of UCS_kaolin_ and UCS_BC_ can be seen in Figure 8a,b, respectively. In each case, the modeled values of the training and validation phases almost go along the observed (experimental) output, which shows the efficiency and accuracy of the formulated MEP models.

For the kaolin soil, the MAE and RMSE of the validation dataset are 6.31% and 7.94% lesser than the training dataset, respectively, and 9.28% and 26.66% lesser, respectively, in the case of BC soil. The improved performance in the testing stage depicts that the proposed MEP models have effectively learned the non-linear relationships among the inputs and response parameters with considerably lower error statistics and higher generalization capability [56,57]. Thus, the proposed model can be used for the prediction of UCS_kaolin_ and UCS_BC_ soil, which will aid in avoiding the heavy testing process.

### 3.3. Model Validity

The validity of the model is an important aspect of the AI modeling process. The model may perform better during the training stage for one set of data, whereas it may yield decreased performance for a new dataset. Therefore, the AI model shall be validated using an unused dataset to investigate the accuracy of the developed model for future applications [46,58,59]. As described in Section 3.2, the developed MEP model was validated using 30% of the experimental data; however, for further validation, the simulated dataset was created to evaluate the effect of contributing parameters on UCS_kaolin_ and UCS_BC_ shown as sensitivity and parametric analysis.

#### Sensitivity Analysis and Parametric Study of MEP Model

The testing of ML-based simulations is critical to ensuring that the recommended models are trustworthy and continue to perform well over a variety of datasets. The goal of sensitivity and parametric research is to confirm the efficacy of the proposed MEP models in terms of their interdependency on physical events [60,61,62]. The sensitivity analysis (SA) of the models on the complete dataset demonstrates how sensitive a generated model is to a change in the input variable in question [57,61,63]. The SA is being used to evaluate the impact of the input factors employed in this study on the anticipated UCS of contaminated soils. 

For a specific independent variable $\left(Y_{i}\right)$, the SA is carried out with Equations (11) and (12) for the overall experimental database considered in the current research. This means that one of the independent variables was changed between its extreme values while keeping the remaining input variables at their average values, and the output was recorded in the form of *f*(*Y_i_*). Next, the second independent variable was changed and the output was monitored.

(11) $$R_{k}=f_{max}\left(Y_{k}\right)-f_{min}\left(Y_{k}\right)$$

(12) $$Relative\;Importance\;\left(\%\right)=SA\;\left(\%\right)=\frac{R_{k}}{\sum_{n}^{j=1}R_{j}}*100$$

**Figure 9:**
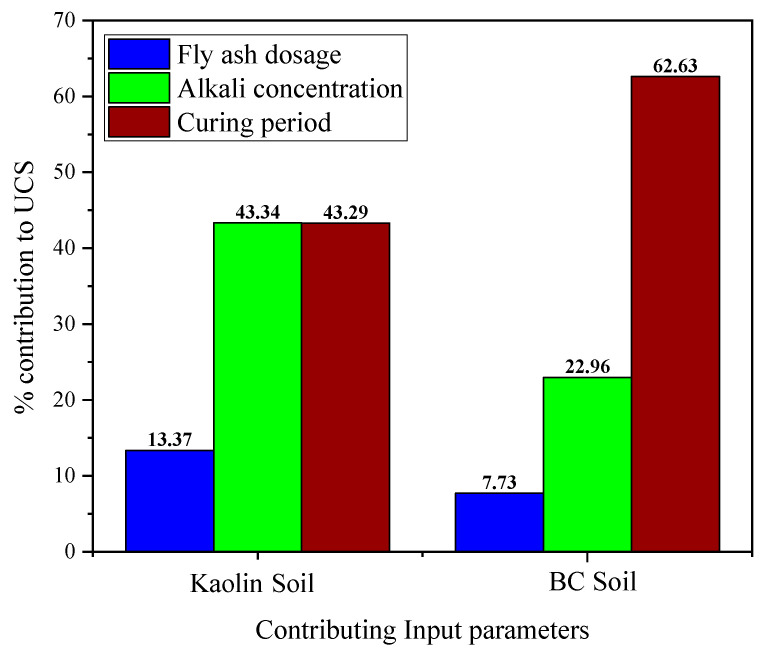
Sensitivity analysis of the developed MEP models: (**a**) kaolin soil; (**b**) BC soil.

$f_{min}\left(Y_{k}\right)$ and $f_{max}\left(Y_{k}\right)$ are the minimum and maximum values of the anticipated results based on *k*th domain of the input variable in the preceding equations, with the remaining inputs kept at their mean. The results of SA can be observed in Figure 9, which shows that the curing period and alkali concentration have almost equal contributions to yielding UCS. The FA dosage contributes 13.37% among the three attributes. In the case of BC soil, the curing period significantly outperforms the other two variables; however, alkali concentration and fly ash dosage also contribute 22.96 and 7.73%, which is an important aspect to consider when investigating FA-incorporated alkali-contaminated soil.

**Figure 10:**
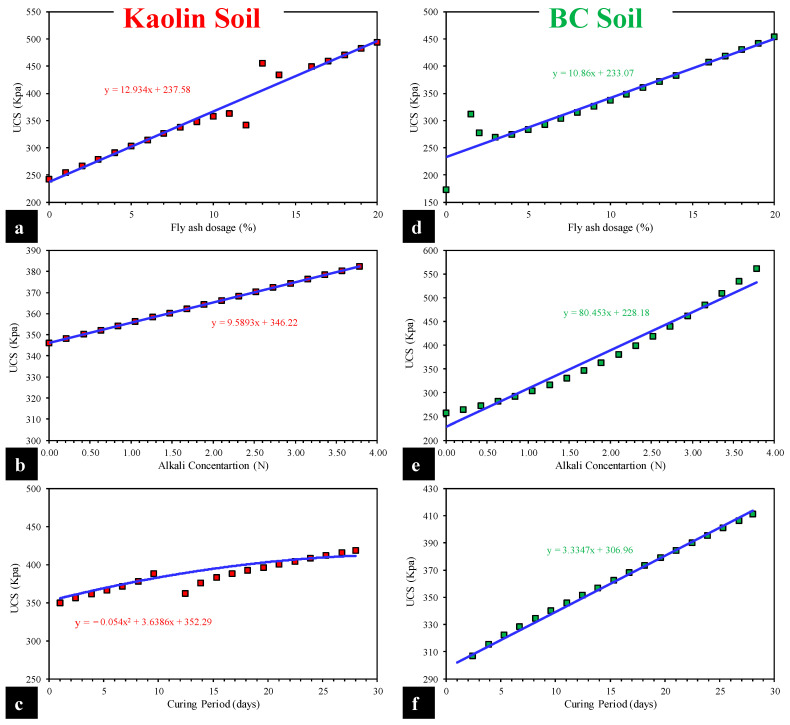
Parametric study of input variables for kaolin and BC soil MEP models: (**a**,**d**) fly ash dosage, (**b**,**e**) alkali concentration, and (**c**,**f**) curing period.

To begin, Figure 10 visually represents the parametric analysis of the inputs used in this work (FA dosage, alkali content, and curing duration) for the prediction of UCS of kaolin and BC soil. The UCS of kaolin soil varies linearly with the amount of fly ash and alkali content, and a second-order polynomial trend is detected for the curing duration. In the case of BC soil, a straightforward linearly rising trend is found for each input (FA dose, alkali content, and curing duration). The increase in UCS of both types of soils with the curing duration is obviously according to the physical process involved, correctly captured by the MEP models, thus validating the models in this respect. Zha et al. [64] found a significant increase in UCS up to 28 days of curing while investigating the stabilization of metal-contaminated soil by alkaline residue. A similar increasing trend in UCS with an increase in curing duration was observed by Fasihnikoutalab et al. [65]. An increase in UCS was also observed with a rise in alkali content and fly ash while investigating alkali-activated geopolymer-incorporated kaolin soil [65]. Therefore, the developed models are deemed validated on the basis of the new experimental dataset and simulated data, which shows the model behavior similar to that of the physical process involved in alkali-activated kaolin and black cotton soil.

## 4. Conclusions

In the present experimental-cum-modeling study, the effect of alkali contamination on the strength characteristics of two clayey soils (i.e., kaolin and BC soil) has been evaluated. The efficiency of FA in remediating the alkali-induced effects was also assessed. Finally, the results were utilized by formulating MEP-based computational prediction models for computing the UCS of both types of the soils (UCS_kaolin_ and UCS_BC_) to overcome the demerits of laborious laboratory testing, cost, and time. The following conclusions can be drawn from the study:

- The inundation of kaolin and BC soils in alkali solution caused the UCS property to decrease. The higher concentrations posed a significant impact in lowering the UCS_kaolin_ and UCS_BC_. On the contrary, the FA treatment of alkali-contaminated soils resulted in a linear increase in the UCS_kaolin_ and UCS_BC_, and an increase of 7-fold was witnessed for the BC soil. Hence, it is concluded that the alkali contamination acted as an activator for a subsequent pozzolanic reaction when FA was incorporated.
- In order to obtain the optimal MEP model for predicting the UCS_kaolin_ and UCS_BC_, a total of 18 trials (each) were undertaken while considering the variation in (a) number of subpopulations, (b) subpopulation size, (c) code length, (d) tournament size, and (e) number of generations. The corresponding performance of all the trials was evaluated using a variety of performance indices, i.e., correlation coefficient and averaged MSE value. The best MEP model (kaolin and BC soil) was achieved in the case of 20 and 70 subpopulations, 1000 and 50 subpopulation size, 100 each code length, 6 each tournament size, 150 and 100 number of generations, 0.9465 and 0.9538 R-value, and 1245 kPa and 4400 kPa averaged MSE value, respectively.
- Simple regression equations developed in this study (Equations (1) and (2)) for kaolin and BC contaminated soils can readily be used to forecast the UCS property. The equations have been generated from relatively high accuracy models evaluated using R, MAE, RMSE, and RSE (0.937, 19.6, 18.271, 0.128 and 0.956, 30, 17.151, 0.108) for the training data of kaolin and BC soils, respectively.
- The generated models were evaluated using parametric and sensitivity analysis as second-level validation. The results obtained from the parametric study manifested a variation in UCS conforming to the literature for kaolin and BC soil with the change in the given input parameters. The sensitivity analysis of kaolin soil showed that curing period and alkali concentration had comparable contributions, followed by the FA dosage, whereas for BC, soil the following increasing trend was observed: curing period > alkali concentration > FA dosage.

## Figures and Tables

**Table 1:** Physical properties of kaolin and BC soils used in the current study.

Property	Kaolin Soil	BC Soil
Specific gravity	2.56	2.65
pH	7.3	7.1
USCS classification	CH	CH
Liquid limit (%)	41	62
Plasticity index (%)	19	28
Optimum moisture content (%)	17	23
Maximum dry density (g/cc)	1.81	1.67

**Table 2:** Chemical composition of fly ash in the current study.

Chemical Constituents	Value (%)
Silica (SiO_2_)	62.9
Alumina (Al_2_O_3_)	21.7
Ferric oxide (Fe_2_O_3_)	4.5
Calcium oxide (CaO)	6.8
Magnesia (MgO)	1.08
Titanium (TiO_2_)	0.06
Potash (K_2_O)	0.04
Sulfur (SO_3_)	0.7
Loss on ignition	2.21

**Table 3:** Experimental database of the input parameters and output parameters in the current study.

S. No.	Fly Ash Dosage (%)	Alkali Concentration (N)	Curing Age (Days)	UCS_BC_ (kPa)	UCS_kaolin_ (kPa)
1	0	0	1	280	255
2	0	0	1	271	261
3	0	0	1	269	259
4	0	0	1	286	275
5	0	0	1	278	268
6	0	0	1	288	277
7	0	0	7	272	262
8	0	0	7	274	264
9	0	0	7	281	270
10	0	0	7	286	275
11	0	0	7	289	278
12	0	0	7	280	269
13	0	0	14	300	265
14	0	0	14	280	262
15	0	0	14	298	279
16	0	0	14	285	266
17	0	0	14	301	281
18	0	0	14	296	277
19	0	0	28	310	270
20	0	0	28	286	267
21	0	0	28	296	277
22	0	0	28	308	288
23	0	0	28	301	281
24	0	0	28	296	276
25	0	1	1	267	236
26	0	1	1	260	231
⁝	⁝	⁝	⁝	⁝	⁝
378	20	4	14	631	851
379	20	4	28	729	998
380	20	4	28	732	987
381	20	4	28	750	996
382	20	4	28	745	1040
383	20	4	28	732	1012
384	20	4	28	740	1004

**Table 4:** Statistical description of input and output parameters used for MEP modeling.

	Fly Ash Dosage (%)	Alkali Concentration (N)	Curing Age (Days)	UCS_BC_ (kPa)	UCS_kaolin_ (kPa)
Minimum	0	0	1	44	119
Maximum	20	4	28	750	1040
Mean	11.25	1.75	12.5	379.51	369.06
Median	12.5	1.5	10.5	365	325.5
SD	7.40	1.48	10.06	109.14	183.30
Kurtosis	−1.1537	−1.1537	−1.1427	1.1374	2.2691
Skewness	−0.4364	0.4364	0.5025	0.8667	1.4534

**Table 5:** Pearson correlation coefficient values for the input parameters and the UCS of alkali-contaminated soils.

	Fly Ash Dosage (%)	Alkali Concentration (N)	Curing (Days)	UCS_kaolin,BC_ (kPa)
Fly ash dosage (%)	1			
Alkali concentration (N)	0	1		
Curing age (days)	0	0	1	
UCS_kaolin_ (kPa)	0.589906	0.508303	0.185189	1
UCS_BC_ (kPa)	0.724809	0.270496	0.321986	1

**Table 6:** Parameter setting for MEP algorithm settings for strength prediction of fly-ash-treated alkali-contaminated soils.

Parameters	Kaolin Soil	BC Soil
Number of subpopulations	20	100
Subpopulation size	1000	2000
Code length	100	80
Crossover probability	0.9	0.9
Crossover type	Uniform	
Mutation probability	0.001	
Tournament size	2	
Operators	0.5	
Variables	0.5	
Constants	0	
Number of generations	150	
Function set	+, −, ×, /	
Terminal set	Problem input	
Replication number	10	
Error measure	Mean squared error	
Problem type	Regression	
Simplified	Yes	
Random seed	0	
Number of runs	10	
Number of threads	1	

**Table 7:** Details of trials undertaken in selecting the best MEP models.

MEP Trial	No. of Subpopulation	Subpopulation Size	Code Length	No. of Generations	Tournament Size	R^2^	R	Avg. MSE	Time (min)
	*Kaolin Soil*								
1	10	100	20	100	2	68.54	82.79	8148	1
2	20					69.28	83.23	7489	1
3	70					66.27	81.41	7177	2
4	100					66.27	81.41	4436	3
5	200					64.57	80.36	5209	6
6	100	500				77.20	87.86	2937	25
7		1000				79.09	88.93	2521	48
8		1500				78.89	88.82	2562	72
9		2000				80.34	89.63	2485	85
10			30			82.60	90.88	2109	130
11			50			83.66	91.47	1951	220
12			80			87.19	93.38	1527	300
13			100			87.65	93.62	1474	429
14				150		88.98	94.33	1455	667
15				200		88.00	93.81	1315	925
16	20	1000		150		87.19	93.37	1551	40
17					4	89.33	94.51	1895	106
**18**					**6**	**89.58**	**94.65**	**1245**	**102**
	*BC Soil*								
1	10	100	20	100	2	72.45	85.12	14,697	1
2	20				2	78.02	88.33	10,980	1
3	70				2	77.81	88.21	11,187	2
4	100				2	77.56	88.07	9578	3
5	200				2	76.39	87.40	9804	8
6	100	500			2	79.19	88.99	8733	23
7		1000			2	80.26	89.59	8486	52
8		1500			2	81.13	90.07	8105	100
9		2000			2	80.88	89.93	8026	145
10			30		2	79.26	89.03	7993	190
11			50		2	78.80	88.77	7256	330
12			80		2	80.55	89.75	6592	357
13			100		2	80.00	89.44	7633	393
**14**			**80**	**150**	**2**	**93.54**	**96.72**	**2220**	**552**
15				200	2	92.19	96.02	2638	549
16	70	500	100	100	2	70.11	83.73	8450	90
17					4	87.66	93.63	5976	110
18					6	90.97	95.38	4400	112

**Table 8:** Performance index values of the final MEP models for alkali-activated soils.

Dataset	Performance Index	Kaolin Soil	BC Soil
Training	R	0.93713	0.95661
	RMSE	18.271	17.151
	MAE	19.6	30.0
	RSE	0.1280	0.1078
	RRMSE	0.0543	0.0564
	NSE	0.8720	0.8922
	ρ	0.0280	0.02882
Testing	R	0.90014	0.96243
	RMSE	21.987	22.995
	MAE	30.5	54.7
	RSE	0.1972	0.0841
	RRMSE	0.0458	0.0441
	NSE	0.8028	0.9159
	ρ	0.0241	0.0225
